# Grip on challenging behaviour: a multidisciplinary care programme for managing behavioural problems in nursing home residents with dementia. Study protocol

**DOI:** 10.1186/1472-6963-11-41

**Published:** 2011-02-21

**Authors:** Sandra A Zwijsen, Martin Smalbrugge, Sytse U Zuidema, Raymond TCM Koopmans, Judith E Bosmans, Maurits W van Tulder, Jan A Eefsting, Debby L Gerritsen, Anne-Margriet Pot

**Affiliations:** 1 Department of Nursing Home Medicine/EMGO + Institute for Health and Care Research, VU Medical Center, Amsterdam, The Netherlands; 2 Department of Primary and Community Care: Centre for Family Medicine, Geriatric Care and Public Health, Radboud University Nijmegen Medical Centre, Nijmegen, the Netherlands; 3 Department of Health Sciences/EMGO + Institute for Health and Care Research, VU University, Amsterdam, The Netherlands; 4 Department of Clinical Psychology, Faculty of Psychological and Educational Sciences, VU University, Amsterdam, The Netherlands; 5 Netherlands Institute of Mental Health and Addiction, Utrecht, The Netherlands

## Abstract

**Background:**

Behavioural problems are common in nursing home residents with dementia and they often are burdensome for both residents and nursing staff. In this study, the effectiveness and cost-effectiveness of a new care programme for managing behavioural problems will be evaluated.

**Methods/Design:**

The care programme is based on Dutch national guidelines. It will consist of four steps: detection, analysis, treatment and evaluation. A stepped wedge design will be used. A total of 14 dementia special care units will implement the care programme. The primary outcome is behavioural problems. Secondary outcomes will include quality of life, prescription rate of antipsychotics, use of physical restraints and workload and job satisfaction of nursing staff. The effect of the care programme will be estimated using multilevel linear regression analysis. An economic evaluation from a societal perspective will also be carried out.

**Discussion:**

The care programme is expected to be cost-effective and effective in decreasing behavioural problems, workload of nursing staff and in increasing quality of life of residents.

**Trial registration:**

The Netherlands National Trial Register (NTR). Trial number: NTR 2141

## Background

Many nursing home (NH) residents with dementia suffer from behavioural problems (BPs) like aggression, apathy and agitation. In a recent Dutch study, BPs were present in 80 percent of the residents [1]. BPs are associated with high costs, diminished quality of life of residents and a high workload for nurses [1-3].

Antipsychotics and physical restraints are frequently used to treat BPs [4,5]. However, the use of antipsychotics may have serious negative side effects like extrapyramidal symptoms and increased risk of stroke [6-8] and the use of restraints may result in decreased functional status and quality of life [9].

Various studies have shown that treatments with less adverse effects can be used to manage BPs as an alternative to antipsychotics and physical restraints. For example, Cohen-Mansfield and colleagues [10] observed a positive effect of individualized psychosocial interventions, such as pain treatment, electronic massagers and individualized music. Furthermore, Livingston et al. found in their review that staff education and psychological and psychosocial treatments were effective [11]. Davison et al. [12] also found a significant decrease in BPs through the use of psychosocial interventions in people with dementia in whom individualised pharmacological treatment failed to work.

In line with these studies, recent professional dementia guidelines emphasise the use of a systematic multidisciplinary approach to treat BPs and stress the importance of psychosocial interventions and staff training [13-16]. They also underline that the use of antipsychotics should be restricted as much as possible. Although these guidelines have been developed in collaboration with long-term care professionals, implementation in actual practice is difficult. Unfortunately, this is also the case in Dutch NHs [17], although the presence of various care disciplines offers excellent conditions for a multidisciplinary approach.

A key problem in implementation of guidelines on BPs seems to be that guidelines do not include a structured, methodology-based approach how to manage BPs [18]. For example, an implementation plan on how different disciplines should work together in managing BPs, is often lacking. Therefore, we developed a care programme entitled: 'Grip on challenging behaviour'. This care-programme, which offers a comprehensible structure of the care processes, is made practically applicable and ready to implement. It is based on the guidelines, fits with daily practice, and describes how new working methods are related to and can be integrated in the present care process following a step-by-step plan.

This paper describes the design of the study that evaluates the effectiveness and cost-effectiveness of this care programme for managing BPs in NH residents with dementia.

## Methods/Design

### Aim

**Figure 1:**
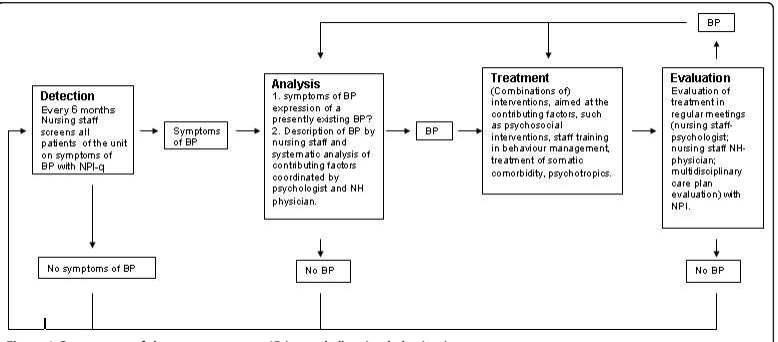
**Components of the care programme 'Grip on challenging behaviour'**.

The aim of this project is to evaluate the effectiveness and cost-effectiveness of a multidisciplinary care programme for managing BPs in NH residents with dementia. The care programme proposes an evidence- and practice-based standardisation of all consecutive steps in the management of BP: detection, analysis, treatment and evaluation (see figure 1). Cooperation between disciplines is also prearranged and structured.

### Intervention

In the first step, the care programme offers a screening tool to detect symptoms of BPs, next to the usual (daily) observation and detection of BPs by nurses. When (symptoms of) BPs are detected, structured forms are used to analyse the behaviour in the next step of the care programme. The nursing staff starts the analysis, after which the elderly care physician and the psychologist continue analysis when necessary. The outcome of the analysis is discussed in pre-arranged multidisciplinary team meetings in which the members of the multidisciplinary team choose the treatment option (or options) they consider appropriate, resulting in a written treatment plan (third step). Psychosocial interventions are first line treatment options and psychotropics or physical restraints should only be used when psychosocial interventions have no or not enough effect. In the fourth step, treatment is evaluated. Standard scales are used for rating BPs when evaluating the effect of interventions. When treatment outcomes are unsatisfactory, alternative treatment options may be chosen and/or a new analysis will be done.

### Design

The care programme will be implemented using a stepped wedge design (Table 1). A stepped wedge design is a type of cross-over design in which different clusters (in this case dementia special care units (SCUs)) cross-over from control-condition to intervention over time [19]. In this study, fourteen participating units are randomly divided over five groups. Four groups consist of three dementia SCUs from three different NHs, one group consists of two dementia SCUs from two different NHs.

**Table 1:** Flow chart of the stepped wedge design (0 = control condition; 1 = intervention).

	T0	T1	T2	T3	T4	T5
Group 1	0	1	1	1	1	1
Group 2	0	0	1	1	1	1
Group 3	0	0	0	1	1	1
Group 4	0	0	0	0	1	1
Group 5	0	0	0	0	0	1

The groups will start with the care programme on six different point in time (T0 through T5)

Six measurement cycles will take place: one measurement cycle every four months during a period of twenty months. The first measurement cycle is a baseline measurement on all participating units. After each measurement cycle, except the last one, a new group will start the intervention. The moment after which measurement cycle a group of units will start is randomised.

A process analysis will be carried out during the study on the actual provision and use of the components of the care programme and on barriers and facilitators of implementation. The process analysis will consist of qualitative interviews with key persons within the NHs.

### Sampling

We calculated the sample-size using the following assumptions: On average, a dementia SCU houses 20 residents. Based on a previous study, we expect that 5% of the residents' (legal) representatives will not give informed consent [1]. We expect no further attrition, because newly admitted residents will replace discharged and deceased residents during the study. For the primary outcome, we assume that our care programme leads to a 10 point decrease of BPs, measured with the Cohen-Mansfield Agitation Inventory (CMAI)[20]. Based on a Dutch study in NH patients [21], we assume a mean Intra Class Correlation Coefficient of 0.1. for clustering of BPs within a unit and a mean score of 47.7 (SD = 16.6) on the CMAI in NH patients with dementia.

Based on these assumptions and a significance level (alpha) of 0.05 and a power (beta) of 0.80, 14 dementia SCUs with 6 measurements are needed in a stepped wedge design.

The participating dementia SCUs will be recruited from NHs that collaborate with the VU University Medical Center (Amsterdam) and the Radboud University Nijmegen, Medical Center. The dementia SCUs participating in this study are not allowed to exchange staff between SCUs, in order to avoid carry-over effects, and thus dilution of the effect.

### Ethical approval

The study protocol was approved by the Medical Ethics Review Committee of the VU University Medical Center. All data will be anonymized and (legal) representatives will have the opportunity to object to the use of data from their relative.

### Measurements

#### Patient characteristics

*Sociodemographic variables *(e.g., age, gender, and length of stay) and the use of physical restraints will be collected from resident charts.

*Severity of dementia *will be determined by elderly care physicians, using the Global Deterioration scale (GDS) [22]. The GDS is a validated seven-point scale that describes seven different stages of dementia ranging from "subjectively and objectively normal" to "severe dementia".

*Data about psychotropic drug use (including antipsychotics) *will be derived from the NH pharmacists' electronic registration system and will be classified according to the Anatomical Therapeutic Chemical (ATC) classification system [23].

*Behavioural problems *will be measured using the Cohen-Mansfield Agitation Inventory (CMAI) and the Neuropsychiatric Inventory - Nursing Home version (NPI-NH). To our knowledge, the CMAI is the only instrument specifically addressing agitation and aggression, with an adequate validity and reliability for the Dutch version [24,25]. The CMAI will be used in primary effect analyses that focus on agitation and aggression, which are the most prevalent and most stressing BPs [4].

The NPI-NH is a version of the Neuropsychiatric Inventory [26] that is adjusted to the NH setting. The questionnaire contains twelve items which each measure the frequency and severity of a neuropsychiatric symptom. It was developed for rating by professional caregivers within institutions [27,28]. The Dutch version proved to be valid and reliable [29].

*Quality of life *of residents will be measured with the Qualidem, a Dutch dementia specific observational quality of life instrument. With this instrument, nursing staff can rate quality of life of the resident over the last week. The Qualidem has nine subscales: Care relationship, Positive affect, Negative effect, Restless tense behaviour, Positive self image, Social relations, Social isolation, Feeling at home and Having something to do. The Qualidem was proven to be valid and reliable, although some items are not applicable to patients with severe dementia (GDS state 7) [30,31].

#### Nursing staff characteristics

Characteristics of the nursing staff (e.g. gender, working experience) are collected through the use of a questionnaire.

Workload of nursing staff will be assessed using the Dutch version of the Maslach Burnout Inventory [32], the Utrechtse Burnout Scale-C [33]. The UBOS measures three components of workload and burnout: emotional exhaustion, depersonalisation and decreased personal accomplishment.

Job satisfaction will be measured using two subscales of the Leiden quality of work questionnaire [34]. The two subscales measure job satisfaction and work and time pressure. The attitude of nursing staff to dementia care will be measured using the approaches to dementia questionnaire (ADQ) [35].

#### Special dementia care unit characteristics

The Special Care Unit Environmental Quality Scale (SCUEQS) is used for the characteristics of the physical environment. The SCUEQS is a summary scale comprised of items from a larger observational instrument (the TESS-NH) which gathers data on the physical environment of a long-term care facility. The eighteen items measure maintenance, cleanliness, safety, lighting, physical appearance/homelikeness, orientation/cuing and noise [36]. In addition information about nursing staff-resident ratio and educational level of nursing staff will be gathered.

### Data analysis

The CMAI-score and the NPI-NH score will be used as a primary outcome. Age, gender, length of stay, dementia severity, prescription of antipsychotics and of other psychotropics will be used as covariates. For the primary and secondary outcome analyses, multilevel linear regression and multilevel logistic regression analyses will be used. These analyses will calculate effects on neuropsychiatric symptoms, quality of life (Qualidem), prescription rate of antipsychotics, workload and job satisfaction of nursing staff and use of physical restraints.

### Economic evaluation

The economic evaluation will be conducted from a societal perspective. We will measure and value all relevant costs, such as costs of the structured care programme, prescription of antipsychotics and hospital admission. Data will be collected using NH registries. Standardised case report forms will be used to measure the time invested by NH staff (e.g. recreational therapist, nursing staff, psychologist, elderly care physician) in both the intervention and the usual care condition. Absence rate of nurses will be retrieved from the participating NHs.

The EuroQol (EQ-5D) proxy version [37] will be used to measure quality adjusted life years (QALYS). Missing data on cost and outcomes will be imputed using multiple imputation according to the MICE algorithm [38].

A cost-effectiveness analysis will be conducted comparing the difference in total mean costs to the difference in effects on BPs; a cost-utility analysis will estimate the incremental costs per QALY. Bootstrapping will be used to estimate uncertainty of the incremental cost-effectiveness ratios (ICERs), which will be presented on cost-effectiveness planes. Cost-effectiveness acceptability curves and net monetary benefits will also be calculated [39]. Sensitivity analysis will include the most important cost-drivers.

## Discussion

The aim of this study is to measure the effectiveness and cost-effectiveness of an evidence- and practice-based care programme for managing BPs in NH residents with dementia. Primary outcome is the effect on prevalence of BP. Secondary outcomes are the effect on quality of life, use of antipsychotics and physical restraints and on workload and job satisfaction of nursing staff. Additionally, an economic evaluation will be carried out.

We assume that implementation of the care programme will result in a decrease of BPs and, subsequently, in an increase of the quality of life of the residents. We also expect lower costs that will most likely be the result of a decrease of behavioural-problem related extra care, a decrease of medication, fewer admissions to hospital and also by a lower absence rate of nursing staff. Implementation is also expected to result in a lower workload and higher job satisfaction among nursing staff.

The chosen design to implement and evaluate the care programme is suitable for our purposes. Not only does the stepped wedge design increase the power of the study by enabling between-groups and within-group analyses, it also ensures that implementation of the care programme occurs in all participating care units, which likely increases motivation for participating in the study [40]. Except for the EQ5D, which is used to calculate QALYs, the chosen outcome parameters are all commonly used in the field of nursing home medicine and are also suitable for the population of severely demented patients [22,24,27,31].

The study has some limitations that should be mentioned. One limitation of the study is that, although data collection will be done by research assistants who are blinded for the trial condition, the NH staff will be aware of receiving the intervention, which may cause bias. To limit this bias, nursing staff will not be informed about the scores on the outcome measures. Another limitation is that we use proxy measures only, which may not be as reliable as patient measures [41]. However, in advancing stages of dementia, cognition and communication decrease, which makes the use of proxy measures inevitable [41]. Nevertheless, the described care programme for managing BP in NH residents with dementia and the chosen stepped wedge design seem very appropriate for our research goals.

## Competing interests

The authors declare that they have no competing interests.

## Authors' contributions

SAZ wrote the paper. MS has designed the study and co-wrote the paper. SUZ has assisted in the design of the study. RK assisted in the design of the study. JB designed the economic evaluation of the study. MT designed the economic evaluation of the study. JE assisted in the design of the study and co-wrote the paper. DG designed the study and co-wrote the paper. AP assisted in the design of the study and co-wrote the paper. All authors have been involved in revising the manuscript of the paper and have given final approval of the publication of the paper.

## Pre-publication history

The pre-publication history for this paper can be accessed here:

http://www.biomedcentral.com/1472-6963/11/41/prepub
